# Supplementary material for: Fatty Acid Profiling as a Tool for Fostering the Traceability of the Halophyte Plant Salicornia ramosissima and Contributing to Its Nutritional Valorization
Source: Plants (Basel). 2024 Feb 16;13(4):545. doi: 10.3390/plants13040545 (PMC10891689; doi:10.3390/plants13040545)
Supplement: Supplementary file 1 [file plants-13-00545-s001.zip › plants-2851418-supplementary.pdf]

Supplementary material

Article

Fatty acid profiling as a tool to foster the traceability of the halophyte plant *Salicornia ramosissima* and contribute to its nutritional valorization

Fernando Ricardo [1*], Ana Carolina Veríssimo [2,3], Elisabete Maciel [2], Maria do Rosário Domingues [2,4*], and Ricardo Calado [1]

Figure S1. Chromatograms of fatty acids identified in the lipid extract of *Salicornia ramosissima* shoots from a) Ria de Aveiro (RAv); b) Mondego estuary (ME); c) Tagus estuary (TE); and d) Ria Formosa (RF). The peaks in detector response correspond to the acquisition time of each fatty acid.

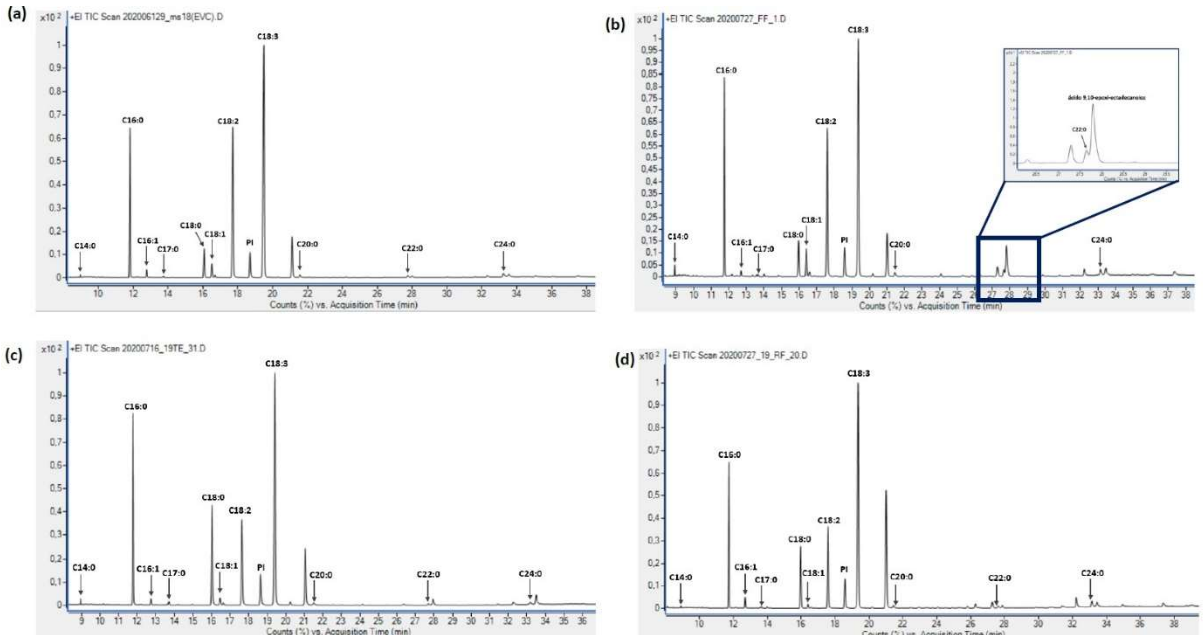

Figure S2. a) Representation of the FAME 9,10-epoxy-octadecanoic acid and its fragments m/z 155 and m/z 199; b) GC-EI mass spectra of 9,10-epoxy-octadecanoic acid; c) GC-EI mass spectrum of FAME 9,10 – epoxy-octadecanoic acid.

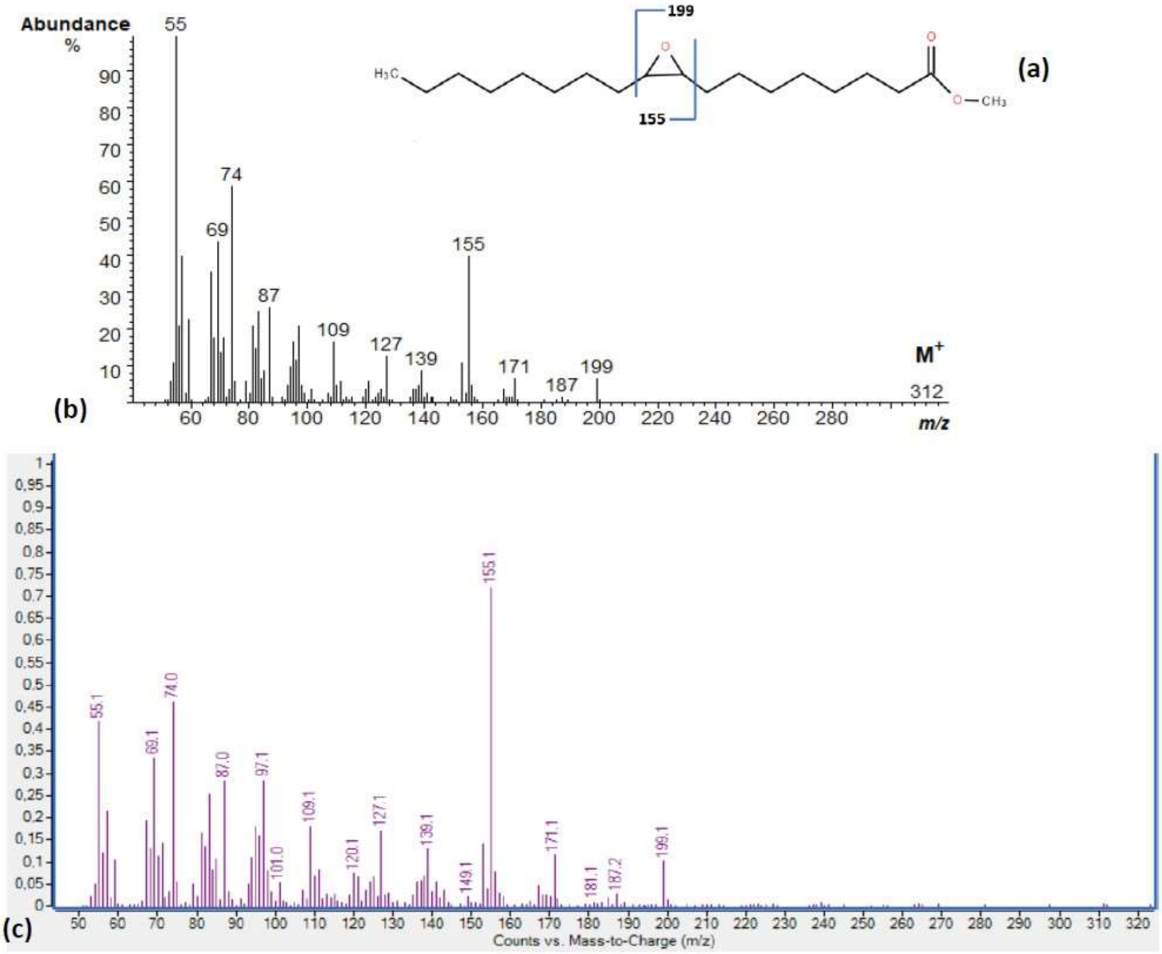

Figure S3. Relative abundances of n-3 and n-6 fatty acids identified in *Salicornia ramosissima* shoots from Ria de Aveiro (RAv), Mondego estuary (ME), Tagus estuary (TE), and Ria Formosa (RF). Different letters indicate significant differences between locations (ANOVA, $p < 0.05$).

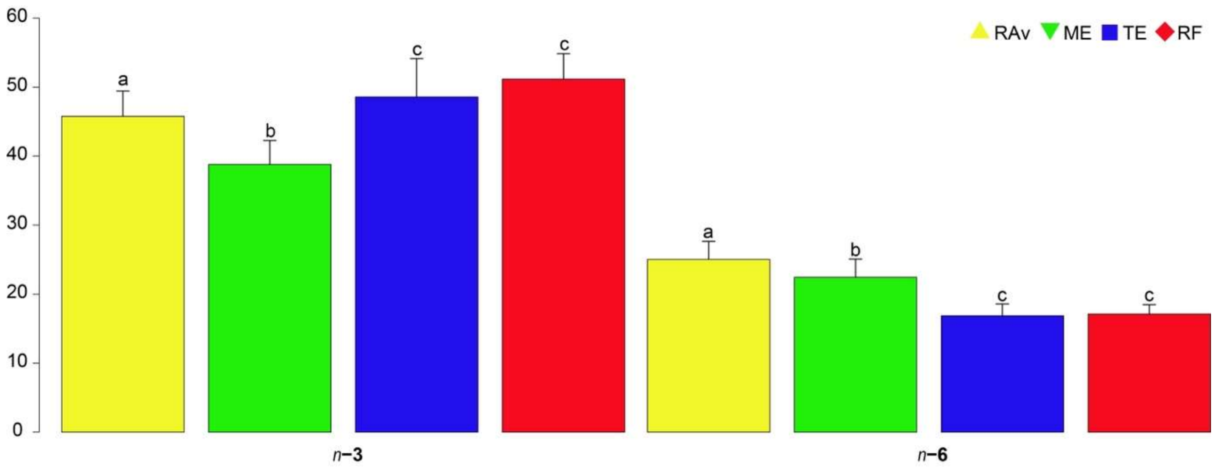

Table S1. P-values resulting from post-hoc comparisons (ANOVA) of the fatty acids identified in the shoots of *Salicornia ramosissima* between the four sampling locations: Ria de Aveiro (RAv), Mondego estuary (ME), Tagus estuary (TE), and Ria Formosa (RF). SFAs – saturated fatty acids; MUFAs – monounsaturated fatty acids; PUFAs – polyunsaturated fatty acids; *n*-3 – *n*-3 fatty acids; *n*-6 – *n*-6 fatty acids; AI - atherogenicity index; TI - thrombogenicity index. Values in bold are lower than 0.05.

	RAv vs ME	RAv vs TE	RAv vs RF	ME vs ET	ME vs RF	TE vs RF
C14:0	**< 0.001**	**0.0039**	**0.005**	**0.002**	**< 0.001**	**< 0.001**
C16:0	**< 0.001**	**< 0.001**	0.215	0.692	**0.002**	0.056
C16:1	**< 0.001**	0.960	**< 0.001**	**< 0.001**	**< 0.001**	**< 0.001**
C17:0	0.755	**0.001**	0.193	**0.017**	**0.017**	**< 0.001**
C18:0	**0.017**	**< 0.001**	0.108	0.382	0.929	0.134
C18:1*n*-9	0.224	**< 0.001**	**< 0.001**	**< 0.001**	**< 0.001**	**0.015**
C18:2*n*-6	0.600	**< 0.001**	**< 0.001**	**< 0.001**	**< 0.001**	0.990
C18:3*n*-3	**0.001**	0.203	**0.002**	**< 0.001**	**< 0.001**	0.337
C20:0	**0.023**	0.911	0.999	**0.003**	**0.035**	0.890
C22:0	**< 0.001**	0.139	0.658	**0.008**	**< 0.001**	0.784
C24:0	**< 0.001**	0.850	**< 0.001**	**< 0.001**	0.880	**< 0.001**
SFAs	**< 0.001**	**< 0.001**	**0.013**	0.924	**0.080**	0.284
MUFAs	0.750	**< 0.001**	**< 0.001**	**< 0.001**	**< 0.001**	0.950
PUFAs	**< 0.001**	**< 0.001**	0.234	0.345	**0.002**	0.217
n-3	**0.001**	0.203	**0.002**	**< 0.001**	**< 0.001**	0.337
n-6	0.600	**< 0.001**	**< 0.001**	**< 0.001**	**< 0.001**	0.990
n-3/*n*-6	0.750	**< 0.001**	**< 0.001**	**< 0.001**	**< 0.001**	0.870
AI	**< 0.001**	**< 0.001**	0.248	0.487	**< 0.001**	**0.021**
TI	**< 0.001**	**0.035**	0.995	0.154	**< 0.001**	0.079

Table S2. Similarity values (ANOSIM) among the fatty acid profiles of *Salicornia ramosissima* shoots sampled in Ria de Aveiro (RAv), Mondego estuary (ME), Tagus estuary (TE), and Ria Formosa (RF).

	R statistic	*p*
RAv vs ME	0.697	0.001
RAv vs TE	0.568	0.001
RAv vs RF	0.874	0.001
ME vs TE	0.763	0.001
ME vs RF	0.895	0.001
TE vs RF	0.576	0.001

Table S3. Classification success of canonical analysis of principal coordinates (CAP) based on the fatty acid profile of *Salicornia ramosissima* collected from four locations along the Portuguese coast: Ria de Aveiro (RAv), Mondego estuary (ME), Tagus estuary (TE), and Ria Formosa (RF).

	Predicted origin				Total samples per site	% successfully classified
	RAv	ME	TE	RF		
RAv	27	2	1	0	30	90.0
ME	3	26	1	0	30	86.7
TE	1	0	29	0	30	96.7
RF	0	0	0	27	27	100.0
Mean						93

Table S4. Classification success of the cross-validation of the canonical analysis of principal coordinates (CAP) based on the fatty acid profile of *Salicornia ramosissima* collected from four locations along the Portuguese coast: Ria de Aveiro (RAv), Mondego estuary (ME), Tagus estuary (TE), and Ria Formosa (RF).

	Predicted site				Total number of samples per site	% of correctly allocated site	% classified			
	RAv	ME	TE	RF			RAv	ME	TE	RF
RAv	15				15	100	100			
ME	6	9			15	60	40	60		
TE	4		11		15	73	27		73	
RF				11	11	100				100

Table S5. Raw data of relative abundance (%) of fatty acids (FAs) present in the shoots of *Salicornia ramosissima* and their respective variation for each FA present in the four locations sampled: Ria de Aveiro (RAv, n = 30); Mondego estuary (ME, n = 30); Tagus estuary (TE, n = 30); and Ria Formosa (RF, n = 27).
