# Supplementary material for: Fatty Acid Profiling as a Tool for Fostering the Traceability of the Halophyte Plant Salicornia ramosissima and Contributing to Its Nutritional Valorization
Source: Plants (Basel). 2024 Feb 16;13(4):545. doi: 10.3390/plants13040545 (PMC10891689; doi:10.3390/plants13040545)
Supplement: Supplementary file 1 [file plants-13-00545-s001.zip › plants-2851418-Table S5.pdf]

Table S5. Raw data of relative abundance (%) of fatty acids (FAs) present in the sh
for each FA present in the four locations sampled: Ria de Aveiro (RAv, n = 30); Mo
Ria Formosa (RF, n = 27).

Samples	C14:0	C16:0	C16:1	C17:0	C18:0	C18:1*n* -9	C18:2*n* -6
**RAv1**	0,2505	18,959	0,9717	0,2541	5,54805	2,304835	25,36313
**RAv2**	0,4374	17,5559	1,1775	0,41	6,05942	2,905931	27,73148
**RAv3**	0,2787	17,9457	0,8825	0,3191	4,04728	3,108981	26,11638
**RAv4**	0,2258	18,004	1,0268	0,2726	3,90664	2,42496	23,36096
**RAv5**	0,2118	17,454	0,8996	0,2119	5,43848	1,953384	21,09708
**RAv6**	0,2887	19,647	0,7768	0,3301	10,3151	2,728328	21,31459
**RAv7**	0,3385	17,1059	0,9947	0,0852	6,8278	2,750374	24,28139
**RAv8**	0,2646	18,2208	1,0375	0,2681	4,42226	2,46171	24,66797
**RAv9**	0,2085	17,3198	1,2283	0,3094	3,98769	2,421711	22,74358
**RAv10**	0,3378	21,5837	1,2117	0,2875	6,25216	2,675633	28,1809
**RAv11**	0,2866	16,0399	0,8742	0,3029	4,90054	3,005967	24,85145
**RAv12**	0,3041	19,8409	0,9598	0,2138	14,4714	2,226053	21,31955
**RAv13**	0,3277	18,1588	1,1471	0,3418	6,7926	2,856474	24,6489
**RAv14**	0,3664	21,5272	1,024	0,2822	6,32556	1,118283	21,87185
**RAv15**	0,2678	16,6238	0,9603	0,2861	3,26287	2,955637	28,97403
**RAv16**	0,3375	18,8841	1,1415	0,3154	3,94287	2,993473	30,85143
**RAv17**	0,156	17,8585	0,9232	0,1739	3,9975	2,36737	25,3523
**RAv18**	0,2182	18,169	0,9228	0,2021	2,75635	2,062332	27,67208
**RAv19**	0,294	17,3419	0,6591	0,259	6,11404	3,301502	20,8176
**RAv20**	0,1683	16,6231	0,8782	0,2402	3,41013	2,633683	26,9415
**RAv21**	0,1853	14,6162	0,8615	0,2	3,27477	2,758132	27,07385
**RAv22**	0,2124	18,0472	0,9116	0,1709	3,4223	2,765408	23,47856
**RAv23**	0,2424	19,2497	0,9741	0,2333	3,55179	3,078711	24,57577
**RAv24**	0,213	16,9527	0,8688	0,2392	2,98122	2,319496	24,13913
**RAv25**	0,1947	16,0686	0,8475	0,2482	4,39473	3,125956	26,6629
**RAv26**	0,215	19,1204	0,9783	0,2285	3,68621	2,675325	25,79908
**RAv27**	0,3241	20,6728	0,9416	0,3219	7,33944	2,470652	24,15631
**RAv28**	0,2286	16,9842	1,0532	0,2352	3,33223	1,651817	21,72264
**RAv29**	0,2042	19,1354	1,1054	0,2482	3,59572	2,990791	28,1578
**RAv30**	0,2433	17,1026	1,0405	0,3095	4,7162	3,158394	27,54908
**ME1**	0,6557	19,8021	0,562	0,2232	4,9088	3,906136	22,45562
**ME2**	0,7455	21,8052	0,449	0,359	10,5638	3,062691	18,60044
**ME3**	1,0484	25,4845	0,4147	0,2499	7,01489	4,221838	17,81308
**ME4**	0,6583	21,3714	0,6354	0,1779	4,84676	2,854413	22,263
**ME5**	0,7033	19,695	0,4735	0,166	4,31696	3,895569	20,655
**ME6**	0,7763	20,7952	0,5543	0,4239	10,8936	4,653374	17,38097
**ME7**	0,4298	18,0745	0,6956	0,1983	3,70403	2,536678	25,43714
**ME8**	0,7062	21,4336	0,8032	0,3252	5,96233	2,608441	23,4647
**ME9**	0,8621	20,9934	0,5652	0,294	5,83374	3,602919	19,99865
**ME10**	0,4837	19,9306	0,5863	0,5828	13,9527	2,633567	19,07432
**ME11**	0,4577	19,5185	0,6471	0,175	4,84975	3,663611	21,93372
**ME12**	0,6805	20,4152	0,6634	0,2391	6,43259	3,520461	21,82715
**ME13**	0,5038	18,4659	0,4221	0,3076	5,28472	2,52433	20,98321

**ME14**	0,5642	18,7399	0,493	0,2179	4,39027	3,059498	23,95496
**ME15**	0,5472	19,0789	0,6369	0,1666	5,37108	2,631681	24,14692
**ME16**	0,4714	20,9689	0,652	0,3457	15,5459	1,938726	19,38988
**ME17**	0,4992	20,5806	0,7766	0,407	7,23592	2,406076	24,85154
**ME18**	0,3727	17,6667	0,5758	0,2067	4,55273	6,935221	28,2839
**ME19**	0,665	22,2846	0,5741	0,1869	5,51295	4,81106	24,09461
**ME20**	0,4085	18,2632	0,7916	0,2758	5,01232	4,527927	26,49985
**ME21**	0,4551	19,9482	0,5474	0,1663	4,89966	2,636704	23,7875
**ME22**	0,3544	18,7221	0,7108	0,2377	4,52532	2,254447	25,0923
**ME23**	0,4288	18,8535	0,72	0,3655	3,95205	3,19785	23,38807
**ME24**	0,3949	18,3176	0	0,2401	4,47477	5,426779	24,73262
**ME25**	0,4774	19,0139	0,6107	0,2965	11,4148	2,611066	21,84152
**ME26**	0,6688	20,2261	0,6082	0,407	6,2007	3,952024	23,56004
**ME27**	0,8385	22,8307	0,7464	0,5054	13,638	3,14056	19,78568
**ME28**	0,4954	19,0053	0,753	0,2897	5,48729	2,295291	24,86016
**ME29**	0,8723	22,18	0,5209	0,2737	5,98671	3,874044	20,93279
**ME30**	0,6464	20,0586	0,6676	0,3093	6,1105	2,557364	22,39439
**TE1**	0,5068	23,5624	1,0447	0,5668	14,0129	1,835858	16,12853
**TE2**	0,3928	18,3599	0,8026	0,4243	6,44218	1,350262	15,60679
**TE3**	0,3737	19,373	0,7763	0,4563	5,48433	1,851908	17,38899
**TE4**	0,3879	20,6896	1,2095	0,3496	5,61504	1,71826	18,39131
**TE5**	0,3743	20,1831	1,1549	0,2003	6,80815	1,91631	17,56924
**TE6**	0,4085	21,6941	1,1659	0,5629	14,8715	1,616342	17,79849
**TE7**	0,2396	19,0245	0,9643	0,1948	7,51205	0,832693	13,62522
**TE8**	0,3804	21,2025	0,9838	0,372	7,18198	1,661725	16,9821
**TE9**	0,3763	20,9528	1,1753	0,487	7,48192	1,801221	19,76225
**TE10**	0,4127	20,989	0,9288	0,5866	12,9884	1,845998	16,60976
**TE11**	0,3076	19,3841	0,8169	0,3097	5,35919	1,620446	16,01439
**TE12**	0,3103	17,4615	0,7939	0,393	6,54945	1,348334	16,98724
**TE13**	0,2053	19,3235	1,0074	0,1718	4,45149	1,074482	16,22861
**TE14**	0,2574	20,8633	0,8306	0,2468	7,55799	1,424125	15,91589
**TE15**	0,3449	22,2305	0,9041	0,2308	10,6237	1,163339	15,30865
**TE16**	0,3817	19,7226	1,0999	0,3751	9,24716	1,445461	17,07918
**TE17**	0,2987	20,8962	1,0018	0,4717	10,4223	1,633338	16,61516
**TE18**	0,3689	21,3383	0,9139	0,4931	14,3902	1,693915	15,44572
**TE19**	0,3578	20,9778	0,8977	0,4187	12,0527	1,568394	17,76567
**TE20**	0,2644	19,0472	1,087	0,4019	4,84676	1,76956	19,12416
**TE21**	0,2935	17,3954	0,7809	0,4478	3,70153	2,094415	18,7196
**TE22**	0,2496	18,3727	0,929	0,4399	3,95619	2,230696	21,04015
**TE23**	0,3253	20,4951	0,8401	0,2024	6,37359	1,853362	16,77882
**TE24**	0,358	19,0243	0,9383	0,3368	9,04097	1,366582	14,23619
**TE25**	0,4872	22,0096	0,8433	0,671	13,3309	2,30938	18,91081
**TE26**	0,407	18,4784	0,8221	0,4481	4,72143	2,354302	18,35138
**TE27**	0,2913	18,1039	1,0978	0,3347	4,29822	1,491577	17,51778
**TE28**	0,3952	21,3353	0,8117	0,5241	15,9336	1,189505	14,57473
**TE29**	0,3952	21,3353	0,8117	0,5241	15,9336	1,189505	14,57473
**TE30**	0,4239	24,0632	1,0343	0,5525	19,2201	2,07549	15,2744
**RF1**	0,2458	21,4474	1,1892	0,3522	15,589	0,65471	14,07059
**RF2**	0,1801	17,9431	1,2185	0,2363	6,46966	0,64125	18,38077
**RF3**	0,1494	18,2217	1,5834	0,1418	5,85077	1,352778	18,2676

**RF4**	0,1428	18,3996	1,6412	0,1746	5,03393	0,956293	17,50727
**RF5**	0,1906	19,0615	1,2595	0,3487	10,4245	1,108395	16,82764
**RF6**	0,1799	19,1423	1,337	0,199	7,584	0,795183	17,30951
**RF7**	0,1032	17,6669	1,372	0,1482	3,9596	0,903854	18,65078
**RF8**	0,1902	19,7699	1,5596	0,3501	16,9555	1,677178	14,57195
**RF9**	0,1152	18,5002	1,7362	0,1735	3,73889	0,925173	17,60355
**RF10**	0,0918	17,642	1,4886	0,134	3,40711	0,748044	18,65808
**RF11**	0,1979	21,4243	1,2707	0,3482	15,7322	0,591021	14,95516
**RF12**	0,1777	20,4732	1,4792	0,2184	8,95598	0,652595	15,86448
**RF13**	0,12	18,0024	1,4053	0,1372	5,04599	0,657946	18,35991
**RF14**	0,0905	18,6933	1,9089	0,1542	4,77973	1,091584	16,99858
**RF15**	0,1781	20,0235	1,7003	0,1254	5,10734	1,636923	17,38559
**RF16**	0,1665	19,7911	1,8598	0,1435	4,70728	0,870063	17,72434
**RF17**	0,1369	17,7805	1,6433	0,185	5,2443	0,728587	18,30634
**RF18**	0,1479	19,2064	2,069	0,148	3,61905	0,960206	17,00871
**RF19**	0,1168	17,8732	1,6963	0,2761	3,8249	0,749867	18,0833
**RF20**	0,1723	18,7725	1,5147	0,2873	11,1002	0,733064	15,76176
**RF21**	0,1648	18,9625	1,8068	0,1922	5,40698	0,861105	16,59878
**RF22**	0,1238	17,7806	1,8783	0,1798	3,55144	0,797851	17,70012
**RF23**	0,178	19,0828	1,5024	0,162	6,57535	0,768235	16,44847
**RF24**	0,196	20,0982	1,4138	0,3369	14,8194	1,103136	15,01567
**RF25**	0,1584	19,0834	1,7016	0,2447	4,22137	0,855473	19,04165
**RF26**	0,1516	18,9293	1,7722	0,2186	6,46351	0,875406	17,29107
**RF27**	0,2312	20,4803	1,6487	0,2172	4,62386	0,710329	18,32639

oots of Salicornia ramosissima and their respective variation
ndego estuary (ME, n = 30); Tagus estuary (TE, n = 30); and

**C18:3*n* -3**	**C20:0**	**C22:0**	**EPOXI (9,10-epoxy-octadecanoic acid)**	**C24:0**
44,38465	0,8451	0,6883	0	0,43065
42,08603	0,3166	0,6104	0	0,70943
45,27984	0,6104	0,7528	0	0,65829
49,27018	0,5908	0,0814	0	0,83577
50,74618	0,4424	0,6854	0	0,85969
43,66258	0,2192	0,0757	0	0,64174
45,85636	0,6129	0,6217	0	0,52513
47,02439	0,3902	0,282	0	0,96035
49,81531	0,6378	0,3301	0	0,99785
36,93291	0,8816	0,4959	0	1,16023
47,35549	0,6831	0,8583	0	0,84140
39,71134	0,3158	0,1728	0	0,46451
43,07664	0,885	0,7735	0	0,99147
45,77725	0,3424	0,5406	0	0,82429
44,0505	0,6099	0,95	0	1,05907
39,31524	0,3698	0,4402	0	1,40840
47,80561	0,4842	0,2103	0	0,67115
47,19103	0,2473	0,2881	0	0,27060
49,04386	0,7873	0,7667	0	0,61499
47,59185	0,3471	0,2812	0	0,88475
48,83885	0,6346	0,7387	0	0,81807
48,55464	0,5988	0,7417	0	1,09647
46,18523	0,5592	0,5094	0	0,84031
50,30025	0,652	0,6765	0	0,65778
47,21402	0,2624	0,2059	0	0,77525
44,99033	0,602	0,8105	0	0,89447
42,39963	0,2786	0,3051	0	0,78990
53,19038	0,4585	0,4823	0	0,66098
42,6847	0,4779	0,6937	0	0,70622
43,32212	0,6013	0,8934	0	1,06355
39,21032	0,7606	0,673	5,80318	1,03942
35,7953	0,5829	0,6442	6,30855	1,08347
37,19955	0,5553	1,0107	3,32258	1,66456
37,51185	0,511	0,6436	7,37462	1,15176
36,83443	0,43	0,5213	11,12909	1,17982
33,91359	0,5898	0,8824	8,15806	0,97853
44,47739	0,7604	0,8498	1,51332	1,32311
40,66791	0,7419	1,3067	0,83171	1,14807
31,97199	0,7204	1,6479	12,15427	1,35542
37,57815	0,5052	0,563	3,36529	0,74438
38,02072	0,4641	0,6821	6,12840	3,45929
36,54886	0,7161	1,2065	6,61845	1,13170
38,65046	0,6826	0,8162	9,71948	1,63954

39,47194	0,7716	1,5433	5,00091	1,79256
38,26455	0,4543	0,642	6,62546	1,43449
39,92013	0,3579	0,4095	0,00000	0,00000
40,42394	0,29	0,6814	0,94864	0,89903
37,2407	0,786	1,1382	0,68181	1,55952
37,0566	0,6052	1,3128	1,21549	1,68065
40,98866	0,8391	1,0192	0,00000	1,37382
44,02106	0,7846	1,0328	0,53354	1,18721
45,20204	0,8154	0,9687	0,00000	1,11692
46,60067	0,4612	0,8343	0,00000	1,19805
42,01123	0,9453	1,1985	0,81209	1,44605
39,5428	0,626	0,8857	1,31178	1,36792
33,73434	0,6136	1,6611	6,76857	1,59953
34,10463	0,6436	1,6155	0,57435	1,57668
40,76496	0,6622	1,1596	3,17182	1,05527
35,80716	0,6325	1,3933	6,02064	1,50591
40,40321	0,7091	1,0046	4,10639	1,03267
40,21428	0,7093	0,806	0	0,61249
54,68423	0,4862	0,7572	0	0,69345
51,36978	0,6623	1,2966	0	0,96687
49,59394	0,654	0,7202	0	0,67063
48,3766	0,6552	1,4789	0	1,28301
39,79157	0,5839	0,7319	0	0,77487
56,95848	0,0915	0,138	0	0,41878
48,94609	0,317	0,7246	0	1,24789
45,93836	0,5046	0,8548	0	0,66548
44,09638	0,4365	0,6269	0	0,47895
54,56539	0,3647	0,5762	0	0,68143
54,79737	0,4409	0,5622	0	0,35578
56,21535	0,153	0,4883	0	0,68074
51,98823	0,1432	0,6488	0	0,12363
48,09487	0,1851	0,2839	0	0,63016
48,20447	0,5832	0,9189	0	0,94230
46,00858	0,8155	0,8582	0	0,97852
43,60914	0,7366	0,6161	0	0,39402
43,09359	1,0077	0,9728	0	0,88715
52,07664	0,3653	0,6053	0	0,41176
53,87481	0,8501	0,989	0	0,85296
50,60149	0,5325	0,889	0	0,75874
50,32736	0,6718	0,9921	0	1,14000
53,91214	0,3422	0,1976	0	0,24682
39,91404	0,2117	0,5443	0	0,76771
51,53226	0,4885	1,1189	0	1,27760
54,53038	0,5812	0,8722	0	0,88101
44,34642	0,185	0,4505	0	0,25395
44,34642	0,185	0,4505	0	0,25395
35,79266	0,5591	0,4874	0	0,51672
44,31615	0,6056	0,5573	0	0,97206
51,91179	0,7219	0,7963	0	1,50030
51,28261	0,705	0,8095	0	1,63538

53,18607	0,5428	0,1162	0	2,29925
48,0166	0,6051	0,8285	0	1,32891
50,05194	0,34	0,9049	0	2,15630
54,31166	0,5781	0,8557	0	1,44994
42,08237	0,541	0,676	0	1,62619
54,19809	0,6665	0,8587	0	1,48410
54,61162	0,4275	0,8853	0	1,90599
43,47516	0,5382	0,5094	0	0,95773
50,40256	0,586	0,5664	0	0,62356
53,87475	0,5262	0,6	0	1,27035
54,35046	0,4767	0,2834	0	1,17264
50,36626	0,6105	0,9555	0	1,91059
52,3218	0,4678	0,7146	0	1,23320
52,97471	0,5421	0,6548	0	1,80350
54,35576	0,5539	0,4909	0	1,44024
54,00997	0,5985	0,7979	0	1,97315
48,9212	0,5521	0,6518	0	1,53307
53,8042	0,5438	0,6165	0	1,04216
55,59048	0,519	0,6002	0	1,27854
52,67148	0,5304	0,7718	0	1,30903
44,8523	0,5445	0,2661	0	1,35395
52,21205	0,5905	0,363	0	1,52783
51,51045	0,5941	0,6772	0	1,51660
51,80642	0,3065	0,3576	0	1,29151
